# The Conceit of the Reformers

**Published:** 1906-02

**Authors:** 


### TEXAS MEDICAL JOURNAL.

AUSTIN, TEXAS.

A MONTHLY JOURNAL OF MEDICINE AND SURGERY.

EDITED AND PUBLISHED BY

F. E. DANIEL, M. D.

Mrs. F. E. DANIEL, Business Manager.

Published Monthly at Austin, Texas. Subscription price $1.00 a year in advance

"I can say that I have no ambition so great as that of being truly esteemed of my fellow men, by rendering myself worthy of their esteem."—*Lincoln at 23.*

### THE CONCEIT OF THE REFORMERS.

*Dear Drs. S. and B.:*

"Suppose the world don't suit you,—nor the way some people do,
Do you think the whole creation should be altered just for you?"

The indiscriminate use of proprietary pharmacal preparations by the medical profession would be an evil which no one denies. The judicious use of certain high-grade preparations known and tried for years and satisfactory, is quite a different thing. It is held that every physician worth mentioning is quite capable of discriminating in the selection of his remedies and to judge of the "ethics" of his practice. They will not be influenced in the least by the dictation of the Octopus and Dr. Frank S. Billings. The undertaking by a coterie of "I-am-holier-than-thou" gentlemen to usurp the privilege of the physician to think for himself and to choose for himself, and to dictate to him, is high-handed and impertinent. The physician will not take up everything advertised and "sampled," but he will still use many elegant and trustworthy preparations which he has found to be reliable, and he knows perfectly well what he is doing when he orders them. If not, he knows perfectly well that the proprietors will furnish the formula on request. The contention of the self-constituted reformers is that the formula should be *published,* and the boycott is used in many shapes to compel it. Were this done scarcely a bottle of a genuine article could be had, for the retail druggist, the doctor's greatest enemy,* would put up "something just as good" every pop. Besides, this undertaking to sift out the proprietaries is the duty of the United States government, and Mr. Yerkes, the Commissioner of Internal Revenue, is at present engaged in the task. I understand that bromidia falls under the ban of the Octopus, and is proscribed. To my certain knowledge the formula of bromidia was published as early as 1879, and I used it in practice.

The medical press, excepting the tentacles of the Octopus, is, so far as I can judge by my exchanges, resenting this boycott business, and the journal that does not do so is the exception. The *New York Medical Record* says, "the medical profession are not children, who require a nurse to see that they do not burn their fingers,"—or fall in the slop pail. If the trustees are wise they will call off the hounds and sit down on Billings.

In this connection I beg to submit the following, respectfully dedicated to *ye* self-righteous reformers of medical ethics:

"A jelly fish† swam in a tropical sea,
And he said: This world consists solely of ME;
Now, all that I learn from the sense of touch
Is the fact of *my feelings* viewed as such.
But to think that these have an external cause,
Is an inference clearly against logical laws,
Again, to suppose, as I have hitherto done,
That there are other jelly fish under the sun,—
Is a poor assumption that can't be backed
By a jot of proof, or a single fact;
In short, like Fitchte, I very much doubt
If there is anything else at all without;
And so, I've come to the plain conclusion,
If the question be only set free from confusion,—
That the universe centers solely in ME,
And if *I were not*,—then, nothing would be."

Just then a shark who was passing by,
Gobbled him up in the twink of an eye,
And he died with a few convulsive twists,
But,—somehow,—the universe still exists.

*See Dr. White's paper in this issue.
†For "jelly fish," read "Octopus."

